# Supplementary material for: Effectiveness of two doses of Euvichol-plus oral cholera vaccine in response to the 2017/2018 outbreak: a matched case–control study in Lusaka, Zambia
Source: BMJ Open. 2022 Nov 11;12(11):e066945. doi: 10.1136/bmjopen-2022-066945 (PMC9660660; doi:10.1136/bmjopen-2022-066945)
Supplement: Author's manuscript [file bmjopen-2022-066945.draft_revisions.pdf]

BMJ Open

BMJ Open is committed to open peer review. As part of this commitment we make the peer review history of every article we publish publicly available.

When an article is published we post the peer reviewers' comments and the authors' responses online. We also post the versions of the paper that were used during peer review. These are the versions that the peer review comments apply to.

The versions of the paper that follow are the versions that were submitted during the peer review process. They are not the versions of record or the final published versions. They should not be cited or distributed as the published version of this manuscript.

BMJ Open is an open access journal and the full, final, typeset and author-corrected version of record of the manuscript is available on our site with no access controls, subscription charges or pay-per-view fees (http://bmjopen.bmj.com).

If you have any questions on BMJ Open's open peer review process please email info.bmjopen@bmj.com

BMJ Open

Effectiveness of two doses of Euvichol-plus oral cholera vaccine in response to the 2017/2018 outbreak: A matched case control study in Lusaka, Zambia

Journal:	*BMJ Open*
Manuscript ID	bmjopen-2022-066945
Article Type:	Original research
Date Submitted by the Author:	27-Jul-2022

Complete List of Authors:	Sialubanje, Cephas; Levy Mwanawasa Medical University, School of Public Health<br>Kapina, Muzala; Zambia National Public Health Institute<br>Chewe, Orbrie; Zambia National Public Health Institute, Surveillance and Disease Intelligence ; Ministry of health Zambia<br>Matapo, Belem; World Health Organization, ;<br>Ngomah, Albertina; Zambia National Public Health Institute, Communication Information & Research<br>Gianetti, Brittany; University of California Berkeley<br>Ngosa, William; Zambia National Public Health Institute, Communication Information & Research<br>Kasonde, Mpanga; Zambia National Public Health Institute, Laboratory System And Networks<br>Musonda, Kunda; Zambia National Public Health Institute, Laboratory Systems and Networks; Ministry of health Zambia,<br>Mulenga, Modest; Tropical Diseases Research Centre<br>Michelo, C; University of Zambia School of Public Health<br>Sinyange, Nyambe; Zambia National Public Health Institute, Epidemic Preparedness & Response<br>Bobo, Patricia; Ministry of health Zambia<br>Zyambo, Khozya; Zambia Ministry of Health<br>Mazyanga, Lucy; Zambia National Public Health Institute<br>Bakyaita, Nathan; World Health Organization<br>Mukonka, Victor M; The Copperbelt University School of Medicine, ; Zambia National Public Health Institute,

Keywords:	Epidemiology < TROPICAL MEDICINE, Public health < INFECTIOUS DISEASES, STATISTICS & RESEARCH METHODS

SCHOLARONE™
Manuscripts

I, the Submitting Author has the right to grant and does grant on behalf of all authors of the Work (as defined in the below author licence), an exclusive licence and/or a non-exclusive licence for contributions from authors who are: i) UK Crown employees; ii) where BMJ has agreed a CC-BY licence shall apply, and/or iii) in accordance with the terms applicable for US Federal Government officers or employees acting as part of their official duties; on a worldwide, perpetual, irrevocable, royalty-free basis to BMJ Publishing Group Ltd ("BMJ") its licensees and where the relevant Journal is co-owned by BMJ to the co-owners of the Journal, to publish the Work in this journal and any other BMJ products and to exploit all rights, as set out in our licence.

The Submitting Author accepts and understands that any supply made under these terms is made by BMJ to the Submitting Author unless you are acting as an employee on behalf of your employer or a postgraduate student of an affiliated institution which is paying any applicable article publishing charge ("APC") for Open Access articles. Where the Submitting Author wishes to make the Work available on an Open Access basis (and intends to pay the relevant APC), the terms of reuse of such Open Access shall be governed by a Creative Commons licence – details of these licences and which Creative Commons licence will apply to this Work are set out in our licence referred to above.

Other than as permitted in any relevant BMJ Author's Self Archiving Policies, I confirm this Work has not been accepted for publication elsewhere, is not being considered for publication elsewhere and does not duplicate material already published. I confirm all authors consent to publication of this Work and authorise the granting of this licence.

For peer review only

Effectiveness of two doses of Euvichol-plus oral cholera vaccine in response to the 2017/2018 outbreak: a matched case control study in Lusaka, Zambia

Cephas Sialubanje[1], Muzala Kapina[2]; Orbrie Chewe[2,3]; Belem B.Matapo[4]; Albertina Ngomah-Moraes[2]; Brittany Gianetti[2,6]; William Ngosa[2]; Mpanga Kasonde[2]; Kunda Musonda[2]; Modest Mulenga[8]; Charles Michelo[7]; Nyambe Sinyange[2]; Patricia Bobo[3]; Khozya Zyambo[3]; Lucy Mazyanga[2]; Nathan Bakyaita[4]; Victor Mukonka[1,2,9]

[1]Levy Mwanawasa Medical University, School of Public Health, Lusaka, Zambia
[2]Zambia National Public Health Institute, Lusaka, Zambia
[3]Ministry of Health, Lusaka, Zambia
[4]World Health Organisation, Lusaka, Zambia
[5]World Health Organisation, Geneva, Switzerland
[6]University of California, Berkeley, California
7School of Public Health, University of Zambia, Lusaka, Zambia
[8]Tropical Diseases Research Centre, Ndola, Zambia
[9]Copperbelt University, School of Medicine, Kitwe, Zambia

Corresponding Author: Cephas Sialubanje
csialubanje@yahoo.com

For peer review only

Abstract

Objectives: Zambia experienced a major cholera outbreak in 2017-18, with more than 5,905 cases reported countrywide, predominantly from the peri-urban slums of Lusaka city. In response to this outbreak, and in line with the World Health Organization (WHO) recommendation, the Zambian government implemented the oral cholera vaccine (OVC) campaign and administered the Euvichol-plus vaccine in the high risk sub-districts of Lusaka. Although OCVs have been shown to be effective in preventing cholera infection in cholera endemic and outbreak settings, the effectiveness of the Euvichol-plus vaccine has not been evaluated in Zambia. This study aimed to determine the effectiveness of two doses of OCV administered during the 2017/18 vaccination campaign.

Methods: Between May and July 2018, we conducted a matched case-control study involving 79 cases and 316 controls following the mass vaccination campaign in the four sub-districts of Lusaka (Chawama, Chipata, Kanyama, and Matero). Matching of controls was based on the place of residence, age and sex. Conditional logistic regression was used for analysis. Adjusted odds ratios (AOR), 95% confidence interval (CI) and vaccine effectiveness (1-AOR) for two doses of Euvichol-plus vaccine and any dose were estimated (p<0.05).

Results: The AOR vaccine effectiveness for two doses of Euvichol-plus OCV was 81.0% (95% CI 66.0%-78.0%; p <0.01). Secondary analysis showed that vaccine effectiveness for any dose was 74.0% (95% CI: 50.0%-86.0%; p <0.01).

Conclusion: These findings show that two doses of Euvichol-plus OCV are effective in a cholera outbreak setting in Lusaka, Zambia. The findings also indicate that two doses are more effective than a single dose and thus support the use of two doses of the vaccine as part of an integrated intervention to cholera control during out breaks.

Word count: 278

Key Words: Cholera: Euvichol-plus; Oral cholera vaccine; Vaccine effectiveness

For peer review only - http://bmjopen.bmj.com/site/about/guidelines.xhtml

Strengths and weaknesses

- Use of a matched case-control study design and collection of data soon after introduction of the vaccine administration minimises confounding and increases internal validity of the study
- Use of a pre-tested electronic data collection tool (ODK) and trained data collectors minimized bias due to measurement error
- Selection of cases from the line list with confirmed diagnosis limits bias due to misclassification
- Use of the vaccination certificate/card to confirm cases' and controls' vaccination status reduced recall and misclassification bias
- Non-probabilistic selection of cases may introduce selection bias and limits generalisation of the study findings

For peer review only - http://bmjopen.bmj.com/site/about/guidelines.xhtml

1. INTRODUCTION

1.1. Background

Cholera is a major public health problem with an increasing global burden [1]. Current estimates indicate that 1.3 billion people are at risk of the disease in endemic countries, resulting in 2.46 million cases and 91 000 deaths worldwide per annum [1, 2]. The low and middle income countries (LMICs) are disproportionately affected and majority (60%) of cases occur in sub-Saharan Africa, with some countries reporting a case fatality rate of 7.5% [1, 2]. Zambia is one of the sub-Saharan African countries with a high burden of cholera, with over 29 cholera outbreaks recorded since 1977 [2] and 34,950 cases reported between 2008 and 2017, the highest number of cases (17,348) having been reported in 2010 from 33 districts around the country [3].

The most recent outbreak in the country (6th October, 2017 to 18th May, 2018) was reported from all the ten provinces of the country and recorded 5, 905 cases [3]. Although all the provinces were affected, past outbreaks have predominantly occurred in the peri-urban slums of Lusaka city, Copperbelt cities and towns, as well as in fishing camps of the Northern, Luapula, Southern, and Central Provinces [3], attributed to poor access to safe water and sanitation facilities.

The Zambian Ministry of Health (MoH) has historically used a multi-sectorial approach that engages various relevant ministries and cooperating partners [4] to implement various interventions including health promotion, provision of safe water and improving sanitation facilities. Though important, these interventions have not been effective in preventing and controlling outbreaks in the country; they are long term and extremely expensive, requiring adequate infrastructure and skilled personnel [5, 6].

To prevent and control cholera outbreaks, the World Health Organization (WHO) recommends use of oral cholera vaccines (OCVs) in conjunction with traditional control measures in cholera endemic areas and during cholera outbreaks [7]. In line with this recommendation, the Zambian government used vaccination programs in response to the 2016 and 2017-18 outbreaks in Lusaka district [8,9] and administered Shanchol vaccine and Euvichol-plus vaccine (uBiologics Co. Ltd., Seoul, Korea), respectively. Both Dukoral (WC-rBS) & Shanchol and Euvichol-plus & mORCVAX (BivWC) OVCs have been found to be effective in preventing cholera infection in cholera endemic and outbreak settings [8, 9]. Further, evidence has shown that two doses of Shanchol provide a cumulative efficacy of 65-74% over a five-year period in cholera endemic settings [10, 11]. The vaccine has been shown to confer even higher short-term protection during cholera outbreaks [12-14]. For example, during the 2016

For peer review only - http://bmjopen.bmj.com/site/about/guidelines.xhtml

outbreak in Lusaka, Zambia, a single dose of Shanchol vaccine provided 88.9% effectiveness in preventing cholera infection [15].

1.2. Objectives

While Euvichol-plus vaccine has been shown to have a similar safety and immunogenicity profile to Shanchol vaccine, its effectiveness has not been ascertained in an outbreak situation in Zambia. The aim of this study, therefore, was to determine the effectiveness of Euvichol-plus vaccine during the 2017/2018 cholera outbreak in Lusaka district, Zambia. This evidence is important to support policy on the use of two doses of the Euvichol-plus vaccine as part of an integrated intervention to cholera control during outbreaks.

2. METHODS

2.1. Study design

Between May and July, 2018 we conducted a retrospective matched case-control study. A matched case-control design allows for controlling of confounding. It also allows use of a relatively small sample, minimizing the need for stratified analysis [16].

2.2. Study setting

The study was conducted in four (Chipata, Matero, Kanyama and Chawama) out of the six sub-districts of Lusaka which were affected by the cholera outbreak. The combined population for the four sub-districts was 1,543,507 [17]. The four districts were selected because that is where the 2018 Euvichol-plus vaccination campaign was conducted. For this purpose, the Zambian government procured a total of 2,070,100 doses of Euvichol-plus vaccine from (WHO) stockpile and implemented two rounds of the OVC campaigns. The first round was carried out from 10 to 20 January 2018, during which 1,317,925 people were vaccinated (as recorded in health facility vaccination reports). Due to logistical challenges, and while waiting for delivery of the complete vaccine stocks of OCV doses, the second round of the campaign was implemented in two phases. Phase one was conducted between 5th and 14th February 2018 and covered two of the four sub-districts (Chawama and Kanyama); phase two was implemented from 18th to 25th April 2018 and included the other two sub-districts (Chipata and Matero).

2.3. Participants and procedures

During the 2017/18 cholera outbreak, seven cholera treatment centres (CTCs) were set up in Kanyama, Chipata, Matero, Chawama, Bauleni, Kalingalinga subdistricts and a central CTC at the national Heroes Stadium. To aid and standardize management of cholera cases in the CTCs, a case definition was established and circulated to all the CTCs. Study participants included cases and controls from the

For peer review only - http://bmjopen.bmj.com/site/about/guidelines.xhtml

cholera treatment centers (CTCs) in the four sub-districts. Cases were selected from the line list of suspected cholera patients admitted to the CTCs. A suspected cholera case was defined as any person who presented to the CTC with at least three episodes of watery diarrhea within the last 24 hours, with or without dehydration or vomiting. On admission to the CTCs, patients suspected to be cholera cases submitted stool samples, which were sent to the microbiology laboratory at the University Teaching Hospital (in Lusaka) for confirmatory analysis using culture. A case was defined as any suspected cholera case with a culture positive result. Controls were recruitment by trained research assistants with help from members of the research team. Controls were matched to cases by place of residence (neighborhood), age and sex, and were systematically selected by visiting the fourth house to the right of a case's home and every consecutive house thereafter. In the event that a potential matched control participant was not available at the selected house at the time of the visit, an appointment was made to return to the house within 48 hours. If the participant was not found after two visits, the next non-enrolled house to the right was visited. Only one control was enrolled per household. If more than one person in a household satisfied the matched control criteria, the person closest in age to the case was enrolled as a control.

2.4. Eligibility criteria

To be eligible for inclusion as a case in the study, a participant needed to:

- Give written informed consent
- Have resided in the study area at the time of the vaccination campaigns
- Have been at least one year of age or older at the time of the vaccination campaigns and had submitted fecal specimen that was found to be culture positive for *V. cholerae* O1

Inclusion criteria for the control participants included

- Any person, aged one year or older at the time of the vaccination campaign
- Residing in the study area
- No history of acute watery diarrhea between January and June 2018

2.5. Sample size estimation

To determine the required sample size for the evaluation and analysis of the Euvichol-plus vaccine effectiveness, the following assumptions were made: 1) 80% of the target population received two doses of OCV; 2) two doses of the vaccine were 75% effective, 3) the study would have 80% power, and 4) 5% margin of error. Based on these assumptions, we determined that the study would need to enroll a minimum of 150 respondents (30 cases and 120 controls; ratio, 1:4). A total of 395

For peer review only - http://bmjopen.bmj.com/site/about/guidelines.xhtml

respondents (that is, 79 cases and 316 controls) were therefore recruited in the study and involved in the analysis.

2.6. Variables

The study variables included the outcome and independent variables as follows:

Outcome variable: cholera status among cases and controls

Independent variables:

a) Demographic information: age, sex, number of children, place of residence
b) Socioeconomic information: level of education, occupation, level of income
c) Vaccination status

2.7. Data collection procedures

An electronic questionnaire, loaded onto a hand-held electronic tablet using the Open Data Kit (ODK) application was used as a data collection tool. Before administering the questionnaire, the research assistant explained the purpose of the study to the participants. Next, informed consent (**patient consent form**) was obtained from the participants who agreed to participate in the study. In addition, in the case of minors, ascent was obtained from guardians or parents. Next, verification of the vaccination exposure was done. To further ascertain vaccination status, participants were asked if they had been vaccinated during the two rounds of the 2018 campaigns and if so, to produce a confirmatory vaccination card. A participant's vaccination exposure was recorded as self-reported if a participant did not possess a vaccination card. Thereafter, data on demographic, socioeconomic and environmental variables (including type of toilet, source of water, level of education, income) was collected and responses recorded onto the ODK. Study participants were either coded zero, one, or two dose vaccine exposure, based on their responses. Vaccine exposure was reclassified as zero dose if a participant reported having received a single dose but spat or vomited it within one to two hours of taking the vaccine, or developed cholera prior to receiving the first dose of vaccine. In addition, vaccine exposure status was reclassified as one dose if a study participant reported having received two doses of vaccine but developed cholera after the first dose was considered effective and prior to administration of a second dose. Multiple doses of vaccine administered to a study participant during the same vaccine campaign (7–10 day period) were treated as a single dose vaccine exposure in the analysis.

2.8. Bias

Possible biases in the study include selection bias, measurement error and confounding due to non-response to vaccination and reinfection. To minimise selection bias, cases were those with a postive

For peer review only - http://bmjopen.bmj.com/site/about/guidelines.xhtml

culture result, and selected from the line list in CTCs. Controls were matched with cases on residence (neighborhood), age and sex. In addition, a group of research team members worked together to select the study sample. To minimise measurement error, research assistants were trained in data collection techniques. To minimise misclassification, a vaccination certificate/card was used to confirm vaccination status. Research assitants worked under supervison from the field supervisors. A pre-tested electronic data collection instrument, ODK was used. Confounding due to place of residence, age and sex were controlled through matching and conditional logistic regression analysis.

2.9. Statistical analysis

Stata/SE version 14 (StataCorp) [18] was used for data analysis. To derive the household asset index, tetrachoric principle component analysis was performed. Next, bivariate analyses were carried out to compare and determine if there were differences in demographic and socioeconomic variables between cases and controls in order to identify potential confounders. Variables found to be significantly associated with case-control status ($p<0.1$) in the bivariate analysis required adjustment in the conditional logistic regression model. Conditional regression analysis was used to determine the association between vaccination status and case and control outcome, accounting for matching variables (place of residence, age and sex). Crude and adjusted odds ratios (AOR) of being vaccinated among the cases and controls, 95% confidence intervals (CI) and vaccine effectiveness were computed. Vaccine effectiveness was calculated as *(1-OR) x 100*. All reported *p*-values and 95% confidence intervals are two-sided ($p<0.05$).

2.10. Patient and Public Involvement

The study design was determined by the research team. Thus, the participants and the public were not directly involved in the conceptualisation and design of the study. However, selection of the study sites was done in consultation with the Ministry of Health and Zambia National Public Health Institute. In addition, selection of study participants was done in collaboration with the provincial and district health managers. First, an inception meeting was held with the ZNPHI and Ministry of Health headquarters staff. In this meeting, the design and objectives of the study were discussed and the four sub-districts selected. Next, pre-field meetings were held with the provincial and district health office staff were held to select the health facilities to be included. Recruitment of participants was done in conjunction with the health facility staff. Finally, a report was written and shared with key stakeholders, including the funding organizations. In addition, a dissemination meeting was held in Lusaka to share the results with the

For peer review only - http://bmjopen.bmj.com/site/about/guidelines.xhtml

World Health Organisation, Ministry of Health and community leaders in the health facilities where the study was conducted.

3. Results

3.1. Participants

A summary of the recruitment algorithm of study participants is shown in figure 1. A total of 5,715 patients were recorded from the six CTCs in Lusaka district, from which 2,761 (48.3%) stool specimens were collected and sent for laboratory confirmation. Of the total number of samples sent for laboratory confirmation, 931 (33.7%) were culture positive for *V.cholerae* O1. Out of the 931 culture positive cholera cases, 97 (10.4%) cases voluntarily agreed to be interviewed and enrolled in the study. These were matched to 388 controls. Upon review of the data, ineligible cases and controls were excluded from the analysis. Additionally, matched cases and controls were excluded from the analysis if their vaccine exposure could not be verified. A study participant's vaccine exposure could not be verified if they did not know whether they had received a dose of the vaccine or not. Cases were ineligible if they developed cholera during the 10 days after receiving the vaccine in the campaign. Thus, 18 cases and their corresponding 72 matched controls who did not meet the inclusion criteria (age, sex and unknown vaccine status) were excluded, leaving 79 cases and 316 controls (total participants=395) in the final analysis (Figure 1).

3.2. Demographic characteristics of the study participants

The demographic characteristics of the study participants are shown in table 1 below. Overall, majority of the respondents were male (58.5) with a mean age of 20.19 (16.26) years for the cases and 20.55 (16.16) years for the controls. Majority of participants (72%) had either primary or secondary education. Most study participants (64%) were classified in the lower two quartiles of the generated household asset index. The average household size was 6 (mean=5.95, SD=2.47) persons. Almost 4 in 5 (79%) study participants used piped water as their primary water source; only 56% treated their drinking water. Most (72%) used a shared pit latrine and 60% reported using soap during handwashing. On average, study participants lived 21.07 (15.53) minutes' walk from the nearest health facility and the most commonly (53%) reported mode of transportation was walking (53%). Less than half (38%) of the study participants reported having consumed food from a market during the outbreak.

Table 1: demographic characteristics

For peer review only - http://bmjopen.bmj.com/site/about/guidelines.xhtml

Characteristic	Cases (n=79)	Controls (n=316)	P value	Total (n=395)
**Age (years)** Mean (SD)	20.19 (16.26)	20.55 (16.16)	0.85	20.47 (16.16)
Sex n (%)			0.76	
Male	45 (57%)	186 (59%)		231 (58.5%)
Female	34 (43%)	130 (41%)		164 (41.5%)
**Education**			0.24	
None	23 (29%)	68 (22%)		91 (23%)
Primary	28 (35%)	131 (41%)		159 (40%)
Secondary	22 (28%)	104 (33%)		126 (32%)
Tertiary	6 (8%)	13 (4%)		19 (5%)
**Occupation**			0.32	
Formal employment	6 (8%)	18 (6%)		24 (6%)
Informal Employment	14 (18%)	58 (18%)		72 (18%)
Unemployed	12 (15%)	54 (17%)		66 (17%)
House wife	8 (10%)	61 (19%)		69 (18%)
Student	18 (23%)	66 (21%)		84 (21%)
Child not in school	21 (26%)	59 (19%)		80 (20%)
**Means of transport**			0.19	
Bicycle	2 (3%)	1 (0.3%)		3 (1%)
Car	23 (29%)	81 (25.6%)		104 (26%)
Public transport	13 (16%)	66 (20.9%)		79 (20%)
Walking	41 (52%)	168 (53.2%)		209 (53%)
**Average time taken to get to nearest health facility (min)**	20.39 (14.36)	21.73(16.14)	0.67	21.07 (15.53)
**Household asset index**			0.28	
0-4	36 (45%)	105 (34%)		141 (36%)
5-7	20 (25%)	87 (28%)		107 (28%)
8-12	17 (22%)	88 (29%)		105 (27%)
12+	6 (8%)	29 (9%)		35 (9%)
**Number of household members**	6.19 (2.85)	5.84 (2.39)	0.39	5.95 (2.47)
**Consumed food at the market**	44 (56%)	202 (64%)	0.18	246 (62%)
No	35 (44%)	114 36%)		149 38%)
Yes				
**Water source**			0.27	
Piped water	57 (72%)	256 (81.0%)		313 (79%)
Well	5 (6%)	11 (3.5%)		16 (4%)
Borehole	17 (22%)	45 (14.3%)		62 (16%)
Rain water	0 (0%)	2 (0.6%)		2 (0.5%)
Bottled water	0 (0%)	2 (0.6%)		2 (0.5%)

For peer review only - http://bmjopen.bmj.com/site/about/guidelines.xhtml

**Water treated before use**<br>No<br>Yes	36 (46%)<br>43 (54%)	139 (44%)<br>177 (56%)	0.80	175 (44%)<br>220 (56%)
**Soap used when washing hands**<br>No<br>Yes	36 (46%)<br>43 (54%)	122 (39%)<br>194 (61%)	0.26	158 (40%)<br>237 (60%)
**Type of toilet**<br>Private pit latrine<br>Shared pit latrine<br>Toilet inside house<br>Other	13 (17%)<br>60 (79%)<br>1 (1%)<br>2 (3%)	63 (21%)<br>214 (70%)<br>22 (7%)<br>6 (2%)	0.20	76 (20%)<br>274 (72%)<br>23 (6%)<br>8 (2%)

3.3. Vaccination status

Out of the total number of participants included in the analysis, 20 (25.3%) cases and 42 (13.3%) controls were unvaccinated (zero dose). Of the total sample, 49 (62.0%) cases and 79 (25%) controls received one dose; 10 (12.7%) cases and 189 (59.8%) controls received two doses, and 59 (74.7%) cases and 268 (84.8%) controls were classified as having received any dose of vaccine (either one or two doses).

Table 2: Vaccination status

	**Vaccination**					
**Variable**	**Case (n=79)**			**Control (n=316)**		
	Yes=59	No=20	P-value	Yes (n=274)	No=42	P-value
	n (%)	n (%)		n(%)	n(%)	
**Sex**			0.594			0.087
Male	34 (57.6)	13.0 (65.00)		166 (60.7)	31 (73.8)	
Female	25 (42,4)	7.0 (35.0)		108 (39.3)	11 (26.2)	
**Age group**			0.032			0.001
<5	19 (32.1)	1 (5.0)		77 (28.1)	5 (11.9)	
5-9	9 (15.3)	1 (5.0)		40 (14.6)	2 (4.8)	
10-19	6 (10.2)	1 (5.0)		23 (8.4)	2 (4.8)	
20-29	10 (16.9)	5 (5.0)		46 (16.8)	16 (38.0)	
30-39	9 (15.3)	7 (5.0)		49 (17.9)	11 (26.2)	
40+	6 (10.2)	5 (25.0)		39 (14.2)	6 (14.3)	
**Occupation**			0.021			0.065
Child not in school	17 (28.8)	1.0 (5.0)		50 (18.3)	7 (16.7)	
Formal employment	5 (8.5)	4 (20.0)		17 (6.2)	6 (14.3)	

For peer review only - http://bmjopen.bmj.com/site/about/guidelines.xhtml

Informal employment	6 (10.2)	7 (35.0)		50 (18.3)	13 (31.0)	
Housewife	7 (11.8)	1 (5.0)		51 (18.6)	3 (7.1)	
Student	14 (23.7)	2 (10.0)		53 (19.3)	5 (11.9)	
Unemployed	6 (10.2)	4 (20.0)		51 (18.6)	8 (19.0)	
Other	4 (6.8)	1 (5.0)		2 (0.7)	0	
**Education**			0.037			0.000
None	26 (33.8)	2 (10.0)		56 (20.5)	8 (19.1)	
Primary	30 (39)	6 (30.0)		119 (43.4)	7 (16.7)	
Secondary	16 (20.8)	9 (45.0)		89 (32.4)	21 (50)	
Tertiary	5 (6.4)	3 (15.0)		10 (3.7)	6 (14.2)	
**Number of people in household**			0.694			0.032
0-4	16(27.1)	7 (35.0)		79 (28.8)	20 (47.6)	
5-7	26 (44.1)	9 (45.0)		136 (49.6)	14 (33.3)	
8-12	14 (23.7)	4 (20.0)		55 (20.1)	8 (19.1)	
12+	3 (5.1)	0.0		4 (1.5)	0 (0.0)	
**Means of transport to health facility**			0.199			0.071
Bicycle	1 (1.7)	1 (5.0)		0	1 (2.4)	
Walking	35 (59.3)	7 (35.0)		143 (52.2)	23 (54.7)	
Car	14 (23.7)	8 (40.0)		70 (25.5)	11 (26.2)	
Public transport	9 (15.3)	4 (20.0)		61 (22.3)	7 (16.7)	
**Drinking treated water**			0.281			0.506
Yes	34 (57.6)	9 (45.0)		156 (56.9)	22 (52.4)	
No	25 (42.4)	11 (55.0)		118 (43.1)	20 (47.6)	

3.4. Bivariate analysis

Bivariate analysis showed no significant differences between the cases and controls (Table 1).

Conditional logistical regression analysis

Conditional logistical regression analysis showed a significant association between two doses of the Euvichol-plus OCV and vaccine protection (AOR=0.19; 95%CI: 0.16-0.28) with a vaccine effectiveness of 81% (95% CI: 72.0%-84.0%; *p*-value <0.01) (Table2). The effectiveness of any (one or more) doses of Euvichol-plus vaccine was 74% (95% CI= 50.0-86.0%; p-value <0.01) (Table 2).

Table 2: Effectiveness of Euvichol vaccination

Number of doses	Cases (n=79)	Controls (n=316)	AOR* (95% CI)	VE	P-value
Any dose	51	268	0.26 (0.14-0.50	74.0% (50-86)	<0.01
2	10	189	0.19 (0.16-0.28)	81.0 (72-84.0)	<0.01

For peer review only - http://bmjopen.bmj.com/site/about/guidelines.xhtml

VE=vaccine effectiveness

*Adjusted for age, sex, place of residence

DISCUSSION

The aim of this study was to determine the effectiveness of Euvichol-plus vaccine administered during a mass OCV campaign as part of control measures following a cholera outbreak in Lusaka. Our findings show that two doses of Euvichol-plus vaccine confer effective protection against cholera and can thus serve as an intervention in the control of cholera outbreaks.

These findings add to the existing evidence on the effectiveness of a two-dose regimen of OCV. They are also consistent with previous studies, for example, a randomised control trial conducted in the Philippines [16] comparing Euvichol and WC-rBS showed that the efficacy of two doses of Euvichol-plus vaccine was non-inferior to that of WC-rBS in adults (80% vs 74%) and children (91% vs 88%)[16]. Thus, our findings suggest that two doses of Euvichol-plus OCV administered during a mass vaccination campaign in response to a cholera outbreak confer effective protection. This finding is also in line with previous observational studies that showed that killed OCVs are protective in endemic and outbreak settings [19, 20]. In an effectiveness study of mass OVC conducted in Beira, Mozambique, Lucas and colleagues [21] found that two doses of WC-rBS vaccine conferred 78% (95% CI: 29-92%) protection when administered during the outbreak. These findings are also consistent with those reported from Tanzania where the WC-rBS vaccine conferred a protection of 79% (95% CI: 47-92%) when administered in cholera endemic areas of Zanzibar [22]. Similarly, two doses of BivWC OCV was found to be effective when deployed in response to cholera outbreaks and areas of high endemicity in Haiti, India, and Guinea, where they conferred 63% (95% CI: 8%–85%), 69% (95% CI: 15-89%), and 87% (95% CI: 57-96%), respectively [23].

Our findings also show that administration of any dose (one or two) conferred significant protection against cholera. This finding is consistent with previous observational studies which reported the effectiveness of a single dose of WC-rBS vaccine [24, 25]. In South Sudan, for example, a study showed that the short-term effectiveness of a single dose of WC-rBS vaccine was 87%, when administered during outbreaks. Similarly, a study conducted in Haiti reported a 79% effectiveness (95% CI: 43-93%) of one dose of OCV [26]

Despite these benefits, our study did not have enough power to determine the long-term effectiveness of the two doses, and thus, it is unclear how long these doses would confer protection against cholera.

For peer review only - http://bmjopen.bmj.com/site/about/guidelines.xhtml

Nevertheless, available evidence shows that vaccine effectiveness wanes over time [26]. A systematic review on the protection against cholera from killed whole-cell OVCs conducted by Bi and colleagues and published in the Lancet found that, although the average effectiveness of two doses of OCV at one year post vaccination was 83%, it decreased to 69% after 2 years. Another study comparing the long-term vaccine effectiveness of two doses against one dose of OCV conducted in Haiti reported that two doses have a significantly higher long-term effectiveness than one dose [25]. It further showed a 76% protection up to four years for two dose vaccine [26]. On the contrary, these authors found that the 79% effectiveness of one dose of OCV observed at 1 year was completely diminished after two years. Thus, these findings clearly show the benefits of administering two doses of OCV. However, further research employing longitudinal study design is required to determine the long-term effectiveness of two doses of OCV in the local context.

Potential limitations of this study should be noted. First, our study findings on the effectiveness of two doses are based on a small sample (10 cases and 189 controls) that received two doses of the Euvichol-plus vaccine. Thus, the study could have been underpowered. Second, our OCV effectiveness was based on an observational epidemiological study design following a reactive vaccination campaign mounted in response to a cholera outbreak. Third, the vaccine exposure status was based on participant self-reports and cards for both cases and controls. This could have introduced information bias and misclassification. In addition, selection of cases from the line list of all suspected cholera patients treated in various CTCs between January and June 2018 might not have represented cholera cases that did not seek care at a health facility.

Despite these limitations, our findings are important as they add to the current evidence on the effectiveness of a two-dose regimen of OCV in the control of cholera outbreaks. In addition, our use of a matched case-control study design, collection of data shortly after the OCV campaign and conditional regression analysis all minimized confounding and increased validity of the study findings. Moreover, selection of cases from the line list with confirmed diagnosis reduced misclassification bias, making the study findings valid.

CONCLUSION

Our findings show that two doses of Euvichol-plus vaccine are effective in the control of the cholera outbreak in Lusaka, Zambia. The findings also show that two doses of the vaccine are more effective than a single dose. These results provide the evidence for and support the use of two doses of the

For peer review only - http://bmjopen.bmj.com/site/about/guidelines.xhtml

vaccine as part of an integrated intervention to cholera control during outbreaks. Nevertheless, it is unclear how long these doses would confer protection against cholera and the long-term effectiveness of the two doses is not well understood. Longitudinal studies are required to determine the long-term effectiveness of two doses of the OCV among the vaccinated populations in the local context. Further research is also required to determine the effectiveness and usefulness of Euvichol-plus vaccine in conferring herd immunity among non-vaccinated individuals during mass immunization and to determine the required minimum coverage. Finally, further research is needed to determine Euvichol-plus vaccine effectiveness among people living with HIV and its usefulness among these populations.

For peer review only

Conflicting interest

All authors declare that they have no conflicting interests in this work

Availability of data and materials

Data are available upon reasonable request from the corresponding author and with permission of the ERES ethics review board.

Costs and payments

There was no cash payment provided to participants for any portion of the study. Participants volunteered their time taken to participate in the study. As a small token of appreciation for their time and opportunity costs, participants received a bottle of water or soft drink, in line with the ERES guideline.

Author contributions

VM, OC, BM, ANM, BG, AK, WN, MK, CS, and MK contributed to the conception of the study, literature search, data collection as well as the drafting of the manuscript including data analysis, figures and results interpretation. KM, AS, CM, NS, PB, KZ, PM, OE, ML and AM contributed to the coordination of the field study activities. DL, EF, LP, NB, KM, CC contributed to the coordination of conception of the study and the literature search. CS, MK, BM, WN contributed to the revision of the manuscript. All authors read and approved the manuscript.

Acknowledgements

We thank Lusaka District Health Management Team who provided field research assistants and study participants. The GTFCC supported the country with OCV stocks and operations funds.

For peer review only - http://bmjopen.bmj.com/site/about/guidelines.xhtml

Funding

The Ministry of Health and World Health Organization funded the study (**award/grant number: N/A**)

Ethics Approval Statement

Ethical approval for the study was obtained from the Excellence in Research, Ethics and Science (ERES) Converge IRB (Ref: ERES/2OO5/29/20). Authority to conduct the study was obtained from the National Health Research Authority. Written informed consent was obtained from all participants in the study

For peer review only

References

1. WHO, 2022. Cholera. Available at: https://www.who.int/news-room/fact-sheets/detail/cholera, accessed on 10th July, 2022
2. Ali M, Lopez AL, You YA, Kim YE, Sah B, Maskery B, Clemens J. The global burden of cholera. Bull World Health Organ. 2012;90(3):209-218A. doi: 10.2471/BLT.11.093427. Epub 2012 Jan 24. PMID: 22461716; PMCID: PMC3314202
3. Mwaba J, Debes AK, Shea P, Mukonka V, Chewe O, Simuyandi M et al. Identification of cholera hotspots in Zambia: A spatiotemporal analysis of cholera data from 2008 to 2017. PLoS Negl Trop Dis 14(4): e0008227. https://doi.org/10.1371/journal.pntd.0008227
4. Olu O, Babaniyi O, Songolo P, Matapo B, Chizema E, Kapin'a-Kanyanga M, Musenga E, Walker O. Cholera Epidemiology in Zambia from 2000 to 2010: Implications for improving Cholera prevention and control strategies in the country. East Afr Med J. 2013;90(10):324-31. Pmid: 26862642.
5. WHO, 2018. Zambia cholera outbreak situation reports available at: https://www.afro.who.int/countries/zambia/situation-reports/cholera, accessed on 10th July, 2022
6. N. Sinyange *et al.*, ‘Cholera Epidemic - Lusaka, Zambia, October 2017-May 2018’, *MMWR Morb. Mortal. Wkly. Rep.*, vol. 67, no. 19, pp. 556–559, May 2018

For peer review only - http://bmjopen.bmj.com/site/about/guidelines.xhtml

7. World Health Organization, 2018. Regional framework for the implementation of the global strategy for cholera prevention and control, 2018–2030: Report of the Secretariat. Regional Office for Africa. Regional Committee for Africa, 68. Available at: . https://apps.who.int/iris/handle/10665/275121, accessed on 10th July, 2022

8. WHO, 2017. Cholera vaccines: WHO position paper – August 201. Available at: https://www.who.int/publications/i/item/who-wer9234-477-500, accessed on 10th July, 2022

9. Bi Q, Ferreras E, Pezzoli L, Legros D, Ivers LC, Date K et al. Protection against cholera from killed whole-cell oral cholera vaccines: a systematic review and meta-analysis. Lancet Infect Dis. 2017;17(10):1080-1088. doi: 10.1016/S1473-3099(17)30359-6

10. Bhattacharya SK, Sur D, Ali M, Kanungo S, You YA, Manna B et al. 5 year efficacy of a bivalent killed whole-cell oral cholera vaccine in Kolkata, India: a cluster-randomised, double-blind, placebo-controlled trial. Lancet Infect Dis. 2013;13 (12):1050-6. doi: 10.1016/S1473-3099(13)70273-1.

11. Qadri F, Wierzba TF, Ali M, Chowdhury F, Khan AI, Saha A et al. Efficacy of a Single-Dose, Inactivated Oral Cholera Vaccine in Bangladesh', *N. Engl. J. Med.*, vol. 374, no. 18, pp. 1723–1732

12. Ferreras E, Chizema-Kawesha E, Blake A, Chewe O, Mwaba J, Zulu G et al. Single-Dose Cholera Vaccine in Response to an Outbreak in Zambia. N Engl J Med. 2018; 378(6):577-579. doi: 10.1056/NEJMc1711583. PMID: 29414267.

13. Luquero FJ, Grout L, Ciglenecki I, Sakoba K, Traore B, Heile M et al. Use of Vibrio cholerae vaccine in an outbreak in Guinea. N Engl J Med. 2014 May 29; 370 (22):2111-20. doi: 10.1056/NEJMoa1312680.

14. Ivers LC, Hilaire IJ, Teng JE, Almazor CP, Jerome JG, Ternier R et al. Effectiveness of reactive oral cholera vaccination in rural Haiti: a case-control study and bias-indicator analysis. The Lancet. Global health, 2015; 33: e162-8

For peer review only - http://bmjopen.bmj.com/site/about/guidelines.xhtml

15. Wierzba TF, Kar SK, Mogasale VV, Kerketta AS, You YA, Baral P et al. Effectiveness of an oral cholera vaccine campaign to prevent clinically-significant cholera in Odisha State, India. Vaccine, 2015; 15; 33 (21):2463-9. doi: 10.1016/j.vaccine.2015.03.073.

16. Verani JR, Baqui AH, Broome CV, Cherian T, Cohen C et al. Case-control vaccine effectiveness studies: Preparation, design, and enrollment of cases and controls. Vaccine. 2017 Jun 5;35(25):3295-3302. doi: 10.1016/j.vaccine.2017.04.037.

17. Central Statistical Office Zambia. 2010 census of population and housing. Available at: https://www.zamstats.gov.zm/phocadownload/2010_Census/2010%20Census%20of%20Population%20National%20Analytical%20Report.pdf, accessed on 1st July, 2022.

18. StataCorp (2015) Stata Statistical Software: Release 14. StataCorp LP, College Station, TX StataCorp LP. - References - Scientific Research Publishing. Available at: https://www.scirp.org/, accessed on 10th July, 2022

19. Azman AS, Parker LA, Rumunu J, Tadesse F, Grandesso F, Deng LL et al. Effectiveness of one dose of oral cholera vaccine in response to an outbreak: a case-cohort study. Lancet Glob Health, 2016; 4(11):e856-e863. doi: 10.1016/S2214-109X(16)30211-X. PMID: 27765293.

20. Baik YO, Choi SK, Olveda RM, Espos RA, Ligsay AD, Montellano MB et al. A randomized, non-inferiority trial comparing two bivalent killed, whole cell, oral cholera vaccines (Euvichol vs Shanchol) in the Philippines. Vaccine. 2015;33 (46):6360-5. doi: 10.1016/j.vaccine.2015.08.075.

21. Lucas ME, Deen JL, von Seidlein L, Wang XY, Ampuero J, Puri M et al. Effectiveness of mass oral cholera vaccination in Beira, Mozambique. N Engl J Med. 2005; 352(8):757-67. doi: 10.1056/NEJMoa043323.

For peer review only - http://bmjopen.bmj.com/site/about/guidelines.xhtml

22. Khatib AM, Ali M, von Seidlein L, Kim DR, Hashim R, Reyburn R et al. Effectiveness of an oral cholera vaccine in Zanzibar: findings from a mass vaccination campaign and observational cohort study. Lancet Infect Dis. 2012; 12(11):837-44. doi: 10.1016/S1473-3099(12)70196-2.

23. Song KR, Lim JK, Park SE, Saluja T, Cho SI, Wartel TA, Lynch J. Oral Cholera Vaccine Efficacy and Effectiveness. Vaccines (Basel). 2021;9(12):1482. doi: 10.3390/vaccines9121482.

24. Schwerdtle P, Onekon CK, Recoche K. A Quantitative Systematic Review and Meta-Analysis of the Effectiveness of Oral Cholera Vaccine as a Reactive Measure in Cholera Outbreaks. Prehosp Disaster Med. 2018 Feb;33(1):2-6. doi: 10.1017/S1049023X17007166.

25. Wierzba TF. Oral cholera vaccines and their impact on the global burden of disease, Human Vaccines & Immunotherapeutics, 2019; 15 (6): 1294-1301, DOI: 10.1080/21645515.2018.1504155

26. Franke MF, Ternier R, Jerome JG, Matias WR, Harris JB, Ivers LC. Long-term effectiveness of one and two doses of a killed, bivalent, whole-cell oral cholera vaccine in Haiti: an extended case-control study. Lancet Glob Health. 2018 Sep;6(9):e1028-e1035. doi: 10.1016/S2214-109X(18)30284-5.

For peer review only

For peer review only - http://bmjopen.bmj.com/site/about/guidelines.xhtml

Figure 1: Summary of participant selection process

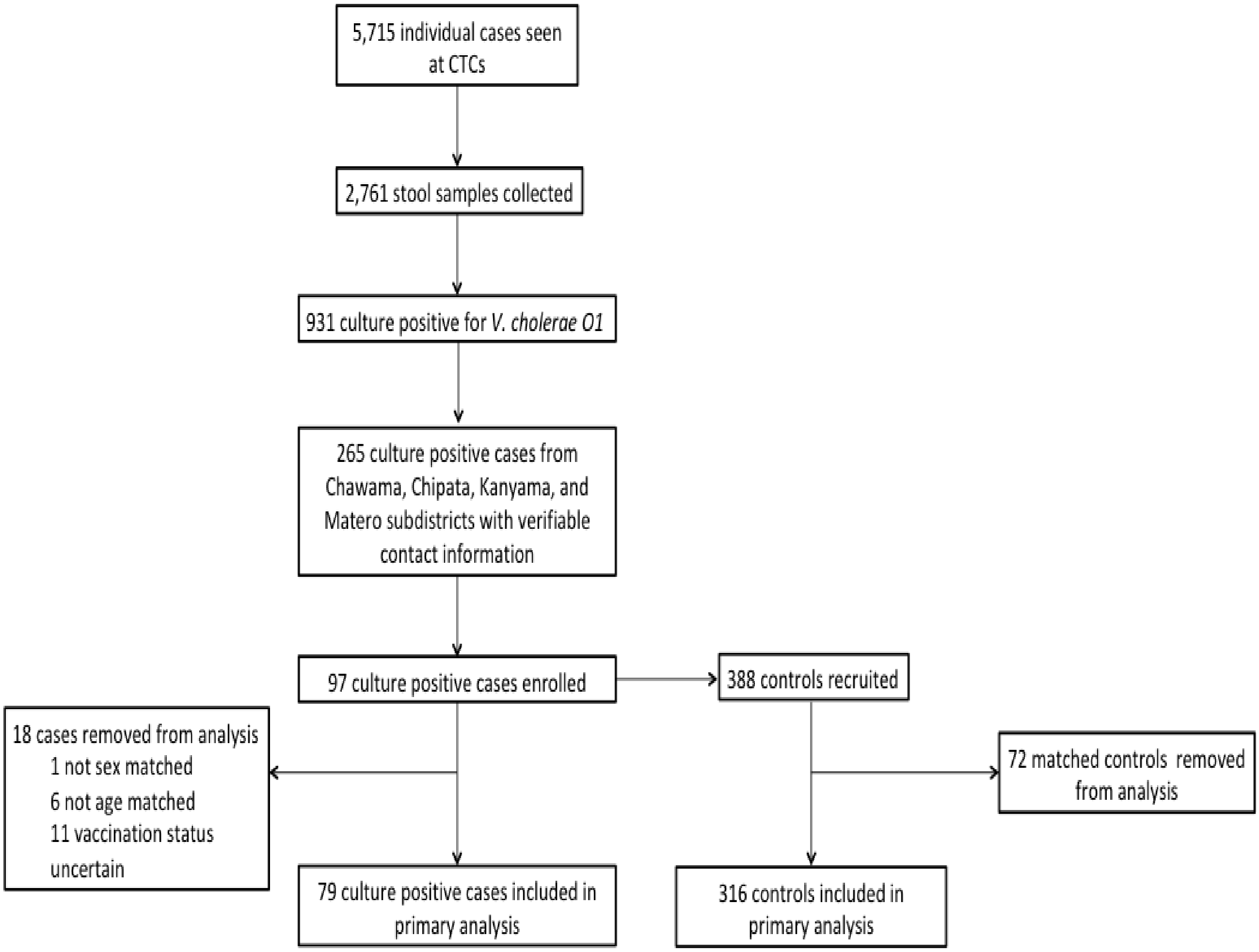

STROBE Statement—Checklist of items that should be included in reports of case-control Studies

**Item**	**Recommendation**	Page
Title and abstract	Indicate the study's design with a commonly used term in the title or the abstract	1
	Provide in the abstract an informative and balanced summary of what was done and what was found	2
Introduction		
Background/rationale	Explain the scientific background and rationale for the investigation being reported	4
Objectives	State specific objectives, including any pre-specified hypotheses	5
Methods		
Study design	Present key elements of study design early in the paper	5
Setting	Describe the setting, locations, and relevant dates, including periods of recruitment, exposure, follow-up, and data collection	5
Participants	Give the eligibility criteria, and the sources and methods of selection of participants. Describe methods of follow-up	5
Study size	Explain how the study size was arrived at	6
Variables	Clearly define all outcomes, exposures, predictors, potential confounders, and effect modifiers. Give diagnostic criteria, if applicable	7
Data sources/ measurement	For each variable of interest, give sources of data and details of methods of assessment (measurement). Describe comparability of assessment methods if there is more than one group	7
Bias	Describe any efforts to address potential sources of bias	8
Statistical methods	Describe all statistical methods, including those used to control for confounding	8
	If applicable, explain how loss to follow-up was addressed	
Patient and public involvement		8
Ethical considerations		9
Results		
Participants	Report numbers of individuals at each stage of study—eg numbers potentially eligible, examined for eligibility, confirmed eligible, included in the study, completing follow-up, and analysed (b) Give reasons for non-participation at each stage Participants (c) Consider use of a flow diagram	9
Descriptive statistics	(a) Give characteristics of study participants (eg demographic, clinical, social) and information on exposures and potential confounders<br>Descriptive data<br>(b) Indicate number of participants with missing data for each variable of interest	10

For peer review only

Outcome data	Report numbers in each exposure category, or summary measures of exposure	12
Main results		13
Discussion		13
Limitations		15
Conclusion		15
References		16

For peer review only

BMJ Open

Effectiveness of two doses of Euvichol-plus oral cholera vaccine in response to the 2017/2018 outbreak: A matched case control study in Lusaka, Zambia

Journal:	*BMJ Open*
Manuscript ID	bmjopen-2022-066945.R1
Article Type:	Original research
Date Submitted by the Author:	19-Oct-2022
Complete List of Authors:	Sialubanje, Cephas; Levy Mwanawasa Medical University, School of Public Health<br>Kapina, Muzala; Zambia National Public Health Institute<br>Chewe, Orbrie; Zambia National Public Health Institute, Surveillance and Disease Intelligence ; Ministry of health Zambia<br>Matapo, Belem; World Health Organization, ;<br>Ngomah, Albertina; Zambia National Public Health Institute, Communication Information & Research<br>Gianetti, Brittany; University of California Berkeley<br>Ngosa, William; Zambia National Public Health Institute, Communication Information & Research<br>Kasonde, Mpanga; Zambia National Public Health Institute, Laboratory System And Networks<br>Musonda, Kunda; Zambia National Public Health Institute, Laboratory Systems and Networks; Ministry of health Zambia,<br>Mulenga, Modest; Tropical Diseases Research Centre<br>Michelo, C; University of Zambia School of Public Health<br>Sinyange, Nyambe; Zambia National Public Health Institute, Epidemic Preparedness & Response<br>Bobo, Patricia; Ministry of health Zambia<br>Zyambo, Khozya; Zambia Ministry of Health<br>Mazyanga, Lucy; Zambia National Public Health Institute<br>Bakyaita, Nathan; World Health Organization<br>Mukonka, Victor M; The Copperbelt University School of Medicine, ; Zambia National Public Health Institute,
**Primary Subject Heading**:	Epidemiology
Secondary Subject Heading:	Epidemiology, Evidence based practice, Infectious diseases, Public health
Keywords:	Epidemiology < TROPICAL MEDICINE, Public health < INFECTIOUS DISEASES, STATISTICS & RESEARCH METHODS

SCHOLARONE™
Manuscripts

I, the Submitting Author has the right to grant and does grant on behalf of all authors of the Work (as defined in the below author licence), an exclusive licence and/or a non-exclusive licence for contributions from authors who are: i) UK Crown employees; ii) where BMJ has agreed a CC-BY licence shall apply, and/or iii) in accordance with the terms applicable for US Federal Government officers or employees acting as part of their official duties; on a worldwide, perpetual, irrevocable, royalty-free basis to BMJ Publishing Group Ltd ("BMJ") its licensees and where the relevant Journal is co-owned by BMJ to the co-owners of the Journal, to publish the Work in this journal and any other BMJ products and to exploit all rights, as set out in our licence.

The Submitting Author accepts and understands that any supply made under these terms is made by BMJ to the Submitting Author unless you are acting as an employee on behalf of your employer or a postgraduate student of an affiliated institution which is paying any applicable article publishing charge ("APC") for Open Access articles. Where the Submitting Author wishes to make the Work available on an Open Access basis (and intends to pay the relevant APC), the terms of reuse of such Open Access shall be governed by a Creative Commons licence – details of these licences and which Creative Commons licence will apply to this Work are set out in our licence referred to above.

Other than as permitted in any relevant BMJ Author's Self Archiving Policies, I confirm this Work has not been accepted for publication elsewhere, is not being considered for publication elsewhere and does not duplicate material already published. I confirm all authors consent to publication of this Work and authorise the granting of this licence.

For peer review only

Effectiveness of two doses of Euvichol-plus oral cholera vaccine in response to the 2017/2018 outbreak: a matched case control study in Lusaka, Zambia

Cephas Sialubanje[1], Muzala Kapina[2]; Orbrie Chewe[2,3]; Belem Matapo[4]; Albertina Moraes Ngomah[2]; Brittany Gianetti[2,6]; William Ngosa[2]; Mpanga Kasonde[2]; Kunda Musonda[2]; Modest Mulenga[8]; Charles Michelo[7]; Nyambe Sinyange[2]; Patricia Bobo[3]; Khozya Zyambo[3]; Lucy Mazyanga[2]; Nathan Bakyaita[4]; Victor Mukonka[1,2,9]

[1]Levy Mwanawasa Medical University, School of Public Health, Lusaka, Zambia

[2]Zambia National Public Health Institute, Lusaka, Zambia

[3]Ministry of Health, Lusaka, Zambia

[4]World Health Organisation, Lusaka, Zambia

[5]World Health Organisation, Geneva, Switzerland

[6]University of California, Berkeley, California

7School of Public Health, University of Zambia, Lusaka, Zambia

[8]Tropical Diseases Research Centre, Ndola, Zambia

[9]Copperbelt University, School of Medicine, Kitwe, Zambia

Corresponding Author: Cephas Sialubanje

csialubanje@yahoo.com

For peer review only

For peer review only - http://bmjopen.bmj.com/site/about/guidelines.xhtml

Abstract

Introduction: Zambia experienced a major cholera outbreak in 2017-18, with more than 5,905 cases reported countrywide, predominantly from the peri-urban slums of Lusaka city. The World Health Organization (WHO) recommends the use of oral cholera vaccines (OCVs) together with traditional control measures, including health promotion, provision of safe water and improving sanitation, in cholera endemic areas and during cholera outbreaks. In response to this outbreak, the Zambian government implemented the OVC campaign and administered the Euvichol-plus vaccine in the high risk sub-districts of Lusaka. Although OCVs have been shown to be effective in preventing cholera infection in cholera endemic and outbreak settings, the effectiveness of the Euvichol-plus vaccine has not yet been evaluated in Zambia. This study aimed to determine the effectiveness of two doses of OCV administered during the 2017/18 vaccination campaign.

Methods: We conducted a matched case-control study involving 79 cases and 316 controls following the mass vaccination campaign in the four sub-districts of Lusaka (Chawama, Chipata, Kanyama, and Matero). Matching of controls was based on the place of residence, age and sex. Conditional logistic regression was used for analysis. Adjusted odds ratios (AOR), 95% confidence interval (CI) and vaccine effectiveness (1-AOR) for two doses of Euvichol-plus vaccine and any dose were estimated (p<0.05).

Results: The AOR vaccine effectiveness for two doses of Euvichol-plus OCV was 81.0% (95% CI 66.0%-78.0%; p <0.01). Secondary analysis showed that vaccine effectiveness for any dose was 74.0% (95% CI: 50.0%-86.0%; p <0.01).

Conclusion: These findings show that two doses of Euvichol-plus OCV are effective in a cholera outbreak setting in Lusaka, Zambia. The findings also indicate that two doses are more effective than a single dose and thus support the use of two doses of the vaccine as part of an integrated intervention to cholera control during outbreaks.

Word count: 297

Key Words: Cholera: Euvichol-plus; Oral cholera vaccine; Vaccine effectiveness

For peer review only - http://bmjopen.bmj.com/site/about/guidelines.xhtml

Strengths and weaknesses

- Use of a matched case-control study design and collection of data soon after introduction of the vaccine minimises confounding and increases internal validity of the study
- Use of a pre-tested electronic data collection tool (ODK) and trained data collectors minimised bias due to measurement error
- Selection of cases from the line list with confirmed diagnosis limits bias due to misclassification
- Use of the vaccination certificate/card to confirm cases' and controls' vaccination status reduced recall and misclassification bias
- A case-control study is not a randomised controlled trial, and evidence from such observational studies is considered relatively weak.

For peer review only

1. INTRODUCTION

1.1. Background

Cholera is a major public health problem with an increasing global burden [1]. Current estimates indicate that 1.3 billion people are at risk of the disease in endemic countries, resulting in 2.46 million cases and 91 000 deaths worldwide per annum [1, 2]. The low and middle income countries (LMICs) are disproportionately affected and majority (60%) of cases occur in sub-Saharan Africa, with some countries reporting a case fatality rate of 7.5% [1, 2]. Zambia is one of the sub-Saharan African countries with a high burden of cholera, with over 29 cholera outbreaks recorded since 1977 [2] and 34,950 cases reported between 2008 and 2017, the highest number of cases (17,348) having been reported in 2010 from 33 districts around the country [3].

The most recent outbreak in the country (6th October, 2017 to 18th May, 2018) was reported from all the ten provinces of the country and recorded 5, 905 cases [3]. Although all the provinces were affected, past outbreaks have predominantly occurred in the peri-urban slums of Lusaka city, Copperbelt cities and towns, as well as in fishing camps of the Northern, Luapula, Southern, and Central Provinces [3], attributed to poor access to safe water and sanitation facilities.

The Zambian Ministry of Health (MoH) has historically used a multi-sectorial approach that engages various relevant ministries and cooperating partners [4] to implement various interventions including health promotion, provision of safe water and improving sanitation facilities. Though important, these interventions have not been effective in preventing and controlling outbreaks in the country; they are long term and extremely expensive, requiring adequate infrastructure and skilled personnel [5, 6].

To prevent and control cholera outbreaks, the World Health Organization (WHO) recommends use of oral cholera vaccines (OCVs) in conjunction with traditional control measures in cholera endemic areas and during cholera outbreaks [7]. In line with this recommendation, the Zambian government used vaccination programs in response to the 2016 and 2017-18 outbreaks in Lusaka district [8,9] and administered Shanchol vaccine and Euvichol-plus vaccine (uBiologics Co. Ltd., Seoul, Korea), respectively. Both Dukoral (WC-rBS) & Shanchol and Euvichol-plus & mORCVAX (BivWC) OVCs have been found to be effective in preventing cholera infection in cholera endemic and outbreak settings [8, 9]. Further, evidence has shown that two doses of Shanchol provide a cumulative efficacy of 65-74% over a five-year period in cholera endemic settings [10, 11]. The vaccine has been shown to confer even higher short-term protection during cholera outbreaks [12-14]. For example, during the 2016

For peer review only - http://bmjopen.bmj.com/site/about/guidelines.xhtml

outbreak in Lusaka, Zambia, a single dose of Shanchol vaccine provided 88.9% effectiveness in preventing cholera infection [15].

1.2. Objectives

While Euvichol-plus vaccine has been shown to have a similar safety and immunogenicity profile to Shanchol vaccine, its effectiveness has not been ascertained in an outbreak situation in Zambia. The aim of this study, therefore, was to determine the effectiveness of Euvichol-plus vaccine during the 2017/2018 cholera outbreak in Lusaka district, Zambia. This evidence is important to support policy on the use of two doses of the Euvichol-plus vaccine as part of an integrated intervention to cholera control during outbreaks.

2. METHODS

2.1. Study design

This was a retrospective matched case-control study. A matched case-control design allows for controlling of confounding. It also allows use of a relatively small sample, minimizing the need for stratified analysis [16].

2.2. Study setting

The study was conducted in four (Chipata, Matero, Kanyama and Chawama) out of the six sub-districts of Lusaka which were affected by the cholera outbreak. The combined population for the four sub-districts was 1,543,507 [17]. The four districts were selected because that is where the 2018 Euvichol-plus vaccination campaign was conducted. For this purpose, the Zambian government procured a total of 2,070,100 doses of Euvichol-plus vaccine from (WHO) stockpile and implemented two rounds of the OVC campaigns. The first round was carried out from 10 to 20 January 2018, during which 1,317,925 people were vaccinated (as recorded in health facility vaccination reports). Due to logistical challenges, and while waiting for delivery of the complete vaccine stocks of OCV doses, the second round of the campaign was implemented in two phases. Phase one was conducted between 5th and 14th February 2018 and covered two of the four sub-districts (Chawama and Kanyama); phase two was implemented from 18th to 25th April 2018 and included the other two sub-districts (Chipata and Matero).

2.3. Participants and procedures

During the 2017/18 cholera outbreak, seven cholera treatment centres (CTCs) were set up in Kanyama, Chipata, Matero, Chawama, Bauleni, Kalingalinga subdistricts and a central CTC at the national Heroes Stadium. To aid and standardize management of cholera cases in the CTCs, a case definition was established and circulated to all the CTCs. Study participants included cases and controls from the

For peer review only - http://bmjopen.bmj.com/site/about/guidelines.xhtml

cholera treatment centers (CTCs) in the four sub-districts. Cases were selected from the line list of suspected cholera patients admitted to the CTCs. A suspected cholera case was defined as any person who presented to the CTC with at least three episodes of watery diarrhea within the last 24 hours, with or without dehydration or vomiting. On admission to the CTCs, patients suspected to be cholera cases submitted stool samples, which were sent to the microbiology laboratory at the University Teaching Hospital (in Lusaka) for confirmatory analysis using culture. A case was defined as any suspected cholera case with a culture positive result. Controls were recruited by trained research assistants with help from members of the research team. Controls were matched to cases by place of residence (neighborhood), age and sex, and were systematically selected by visiting the fourth house to the right of a case's home and every consecutive house thereafter. In the event that a potential matched control participant was not available at the selected house at the time of the visit, an appointment was made to return to the house within 48 hours. If the participant was not found after two visits, the next non-enrolled house to the right was visited. Only one control was enrolled per household. If more than one person in a household satisfied the matched control criteria, the person closest in age to the case was enrolled as a control.

2.4. Eligibility criteria

To be eligible for inclusion as a case in the study, a participant needed to:

- Give written informed consent
- Have resided in the study area at the time of the vaccination campaigns
- Have been at least one year of age or older at the time of the vaccination campaigns and had submitted fecal specimen that was found to be culture positive for *V. cholerae* O1

Inclusion criteria for the control participants included

- Any person, aged one year or older at the time of the vaccination campaign
- Residing in the study area at the time of the vaccination campaigns
- No history of acute watery diarrhea between January and June 2018

2.5. Sample size estimation

To determine the required sample size for the evaluation and analysis of the Euvichol-plus vaccine effectiveness, the following assumptions were made: 1) 80% of the target population received two doses of OCV; 2) two doses of the vaccine were 75% effective, 3) the study would have 80% power, and 4) 5% margin of error. Based on these assumptions, we determined that the study would need to

For peer review only - http://bmjopen.bmj.com/site/about/guidelines.xhtml

enroll a minimum of 150 respondents (30 cases and 120 controls; ratio, 1:4). A total of 395 respondents (that is, 79 cases and 316 controls) were therefore recruited in the study and involved in the analysis.

2.6. Variables

The study variables included the outcome and independent variables as follows:

Outcome variable: cholera status among cases and controls

Independent variables:

a) Demographic information: age, sex, number of children, place of residence
b) Socioeconomic information: level of education, occupation, level of income
c) Vaccination status

2.7. Data collection procedures

An electronic questionnaire, loaded onto a hand-held electronic tablet using the Open Data Kit (ODK) application was used as a data collection tool. Before administering the questionnaire, the research assistant explained the purpose of the study to the participants. Next, informed consent (**patient consent form**) was obtained from the participants who agreed to participate in the study. In addition, in the case of minors, ascent was obtained from guardians or parents. Next, verification of the vaccination exposure was done. To further ascertain vaccination status, participants were asked if they had been vaccinated during the two rounds of the 2018 campaigns and if so, to produce a confirmatory vaccination card. A participant's vaccination exposure was recorded as self-reported if a participant did not possess a vaccination card. Thereafter, data on demographic, socioeconomic and environmental variables (including type of toilet, source of water, level of education, income) was collected and responses recorded onto the ODK. Study participants were either coded zero, one, or two dose vaccine exposure, based on their responses. Vaccine exposure was reclassified as zero dose if a participant reported having received a single dose but spat or vomited it within one to two hours of taking the vaccine, or developed cholera prior to receiving the first dose of vaccine. In addition, vaccine exposure status was reclassified as one dose if a study participant reported having received two doses of vaccine but developed cholera after the first dose was considered effective and prior to administration of a second dose. Multiple doses of vaccine administered to a study participant during the same vaccine campaign (7–10 day period) were treated as a single dose vaccine exposure in the analysis.

2.8. Bias

Possible biases in the study include selection bias, measurement error and confounding due to non-response to vaccination and reinfection. To minimise selection bias, cases were those with a postive

For peer review only - http://bmjopen.bmj.com/site/about/guidelines.xhtml

culture result, and selected from the line list in CTCs. Controls were matched with cases on residence (neighborhood), age and sex. In addition, a group of research team members worked together to select the study sample. To minimise measurement error, research assistants were trained in data collection techniques. To minimise misclassification, a vaccination certificate/card was used to confirm vaccination status. Research assitants worked under supervison from the field supervisors. A pre-tested electronic data collection instrument, ODK was used. Confounding due to place of residence, age and sex were controlled through matching and conditional logistic regression analysis.

2.9. Statistical analysis

Stata/SE version 14 (StataCorp) [18] was used for data analysis. To derive the household asset index, tetrachoric principle component analysis was performed. Descriptive statistics were done to summarise demographic and socioeconomic characteristics of participants (**table 1**). Next, bivariate analyses were carried out to compare and determine if there were differences in demographic and socioeconomic variables between cases and controls in order to identify potential confounders (**table 2**). Variables found to be significantly associated with case-control status ($p<0.1$) in the bivariate analysis required adjustment in the conditional logistic regression model (**table 3**). Conditional regression analysis was used to determine the association between vaccination status and case and control outcome, accounting for matching variables (place of residence, age and sex). Crude and adjusted odds ratios (AOR) of being vaccinated among the cases and controls, 95% confidence intervals (CI) and vaccine effectiveness were computed. Vaccine effectiveness was calculated as *(1-OR) x 100*. All reported *p*-values and 95% confidence intervals are two-sided ($p<0.05$).

2.10. Patient and Public Involvement

The study design was determined by the research team. Participants and the public were not directly involved in the conceptualisation and design of the study. However, selection of the study sites was done in consultation with stakeholders from the Ministry of Health and Zambia National Public Health Institute. Selection of study participants was done in collaboration with the provincial and district health managers. A dissemination meeting was held in Lusaka and study findings shared with key stakeholders, including the World Health Organisation, Ministry of Health, and community leaders in the health facilities where the study was conducted. A final report was also written and shared with the funding organization.

3. Results

For peer review only - http://bmjopen.bmj.com/site/about/guidelines.xhtml

3.1. Participants

A summary of the recruitment algorithm of study participants is shown in figure 1. A total of 5,715 patients were recorded from the six CTCs in Lusaka district, from which 2,761 (48.3%) stool specimens were collected and sent for laboratory confirmation. Of the total number of samples sent for laboratory confirmation, 931 (33.7%) were culture positive for *V.cholerae* O1. Out of the 931 culture positive cholera cases, only 265 (28.5%) cases had verifiable contact information and were contacted for possible participation in the study; 97 (10.4%) cases voluntarily agreed and accepted to be interviewed and enrolled in the study. These were matched to 388 controls. Upon review of the data, cases and controls were found ineligible and did not meet the inclusion criteria (age, sex and vaccine status) were excluded from the analysis. Matched cases and controls were excluded from the analysis if their vaccine exposure could not be verified. A study participant's vaccine exposure could not be verified if they did not know whether they had received a dose of the vaccine or not. Cases were also excluded if they developed cholera during the 10 days after receiving the vaccine in the campaign. Thus, 18 cases and their corresponding 72 matched controls did not meet the inclusion criteria (age, sex and unknown vaccine status) and were excluded, leaving a total of 79 cases and 316 controls (n=395) in the final analysis (Figure 1).

3.2. Demographic characteristics of the study participants

The demographic characteristics of the study participants are shown in table 1 below. Overall, majority of the respondents were male (58.5) with a mean age of 20.19 (16.26) years for the cases and 20.55 (16.16) years for the controls. Majority of participants (72%) had either primary or secondary education. Most study participants (64%) were classified in the lower two quartiles of the generated household asset index. The average household size was 6 (mean=5.95, SD=2.47) persons. Almost 4 in 5 (79%) study participants used piped water as their primary water source; only 56% treated their drinking water. Most (72%) used a shared pit latrine and 60% reported using soap during handwashing. On average, study participants lived 21.07 (15.53) minutes' walk from the nearest health facility and the most commonly (53%) reported mode of transportation was walking (53%). Less than half (38%) of the study participants reported having consumed food from a market during the outbreak.

Table 1: Demographic characteristics

Characteristic	Cases (n=79)	Controls (n=316)	P value	Total (n=395)
**Age (years)**<br>Mean (SD)	20.19 (16.26)	20.55 (16.16)	0.85	20.47 (16.16)
Sex n (%)<br>Male	 45 (57%)	 186 (59%)	0.76	 231 (58.5%)

Female	34 (43%)	130 (41%)		164 (41.5%)
**Education**			0.24	
None	23 (29%)	68 (22%)		91 (23%)
Primary	28 (35%)	131 (41%)		159 (40%)
Secondary	22 (28%)	104 (33%)		126 (32%)
Tertiary	6 (8%)	13 (4%)		19 (5%)
**Occupation**			0.32	
Formal employment	6 (8%)	18 (6%)		24 (6%)
Informal Employment	14 (18%)	58 (18%)		72 (18%)
Unemployed	12 (15%)	54 (17%)		66 (17%)
House wife	8 (10%)	61 (19%)		69 (18%)
Student	18 (23%)	66 (21%)		84 (21%)
Child not in school	21 (26%)	59 (19%)		80 (20%)
**Means of transport**			0.19	
Bicycle	2 (3%)	1 (0.3%)		3 (1%)
Car	23 (29%)	81 (25.6%)		104 (26%)
Public transport	13 (16%)	66 (20.9%)		79 (20%)
Walking	41 (52%)	168 (53.2%)		209 (53%)
**Average time taken to get to nearest health facility (min)**	20.39 (14.36)	21.73(16.14)	0.67	21.07 (15.53)
**Household asset index**			0.28	
0-4	36 (45%)	105 (34%)		141 (36%)
5-7	20 (25%)	87 (28%)		107 (28%)
8-12	17 (22%)	88 (29%)		105 (27%)
12+	6 (8%)	29 (9%)		35 (9%)
**Number of household members**	6.19 (2.85)	5.84 (2.39)	0.39	5.95 (2.47)
**Consumed food at the market**			0.18	
No	44 (56%)	202 (64%)		246 (62%)
Yes	35 (44%)	114 36%)		149 38%)
**Water source**			0.27	
Piped water	57 (72%)	256 (81.0%)		313 (79%)
Well	5 (6%)	11 (3.5%)		16 (4%)
Borehole	17 (22%)	45 (14.3%)		62 (16%)
Rain water	0 (0%)	2 (0.6%)		2 (0.5%)
Bottled water	0 (0%)	2 (0.6%)		2 (0.5%)
**Water treated before use**			0.80	
No	36 (46%)	139 (44%)		175 (44%)
Yes	43 (54%)	177 (56%)		220 (56%)

For peer review only

**Soap used when washing hands**			0.26	
No	36 (46%)	122 (39%)		158 (40%)
Yes	43 (54%)	194 (61%)		237 (60%)
**Type of toilet**			0.20	
Private pit latrine	13 (17%)	63 (21%)		76 (20%)
Shared pit latrine	60 (79%)	214 (70%)		274 (72%)
Toilet inside house	1 (1%)	22 (7%)		23 (6%)
Other	2 (3%)	6 (2%)		8 (2%)

3.3. Vaccination status

Out of the total number of participants included in the analysis, 20 (25.3%) cases and 42 (13.3%) controls were unvaccinated (zero dose). Of the total sample, 49 (62.0%) cases and 79 (25%) controls received one dose; 10 (12.7%) cases and 189 (59.8%) controls received two doses, and 59 (74.7%) cases and 268 (84.8%) controls were classified as having received any dose of vaccine (either one or two doses).

Table 2: Vaccination status

	Vaccination					
**Variable**	**Case (n=79)**			**Control (n=316)**		
	Yes=59	No=20	P-value	Yes (n=274)	No=42	P-value
	n (%)	n (%)		n(%)	n(%)	
**Sex**			0.594			0.087
Male	34 (57.6)	13.0 (65.00)		166 (60.7)	31 (73.8)	
Female	25 (42,4)	7.0 (35.0)		108 (39.3)	11 (26.2)	
**Age group**			0.032			0.001
<5	19 (32.1)	1 (5.0)		77 (28.1)	5 (11.9)	
5-9	9 (15.3)	1 (5.0)		40 (14.6)	2 (4.8)	
10-19	6 (10.2)	1 (5.0)		23 (8.4)	2 (4.8)	
20-29	10 (16.9)	5 (5.0)		46 (16.8)	16 (38.0)	
30-39	9 (15.3)	7 (5.0)		49 (17.9)	11 (26.2)	
40+	6 (10.2)	5 (25.0)		39 (14.2)	6 (14.3)	
**Occupation**			0.021			0.065
Child not in school	17 (28.8)	1.0 (5.0)		50 (18.3)	7 (16.7)	
Formal employment	5 (8.5)	4 (20.0)		17 (6.2)	6 (14.3)	
Informal employment	6 (10.2)	7 (35.0)		50 (18.3)	13 (31.0)	
Housewife	7 (11.8)	1 (5.0)		51 (18.6)	3 (7.1)	
Student	14 (23.7)	2 (10.0)		53 (19.3)	5 (11.9)	
Unemployed	6 (10.2)	4 (20.0)		51 (18.6)	8 (19.0)	
Other	4 (6.8)	1 (5.0)		2 (0.7)	0	

For peer review only - http://bmjopen.bmj.com/site/about/guidelines.xhtml

**Education**			0.037			0.000
None	26 (33.8)	2 (10.0)		56 (20.5)	8 (19.1)	
Primary	30 (39)	6 (30.0)		119 (43.4)	7 (16.7)	
Secondary	16 (20.8)	9 (45.0)		89 (32.4)	21 (50)	
Tertiary	5 (6.4)	3 (15.0)		10 (3.7)	6 (14.2)	
**Number of people in household**			0.694			0.032
0-4	16(27.1)	7 (35.0)		79 (28.8)	20 (47.6)	
5-7	26 (44.1)	9 (45.0)		136 (49.6)	14 (33.3)	
8-12	14 (23.7)	4 (20.0)		55 (20.1)	8 (19.1)	
12+	3 (5.1)	0.0		4 (1.5)	0 (0.0)	
**Means of transport to health facility**			0.199			0.071
Bicycle	1 (1.7)	1 (5.0)		0	1 (2.4)	
Walking	35 (59.3)	7 (35.0)		143 (52.2)	23 (54.7)	
Car	14 (23.7)	8 (40.0)		70 (25.5)	11 (26.2)	
Public transport	9 (15.3)	4 (20.0)		61 (22.3)	7 (16.7)	
**Drinking treated water**			0.281			0.506
Yes	34 (57.6)	9 (45.0)		156 (56.9)	22 (52.4)	
No	25 (42.4)	11 (55.0)		118 (43.1)	20 (47.6)	

3.4. Bivariate analysis

Bivariate analysis showed no significant differences between the cases and controls with regard to sex, occupation, means of transport and drinking treated water. Significant differences between the two groups were noted with regard to education, number of people in the households (table 2).

3.5. Conditional logistical regression analysis

Conditional logistical regression analysis showed a significant association between two doses of the Euvichol-plus OCV and vaccine protection (AOR=0.19; 95%CI: 0.16-0.28) with a vaccine effectiveness of 81% (95% CI: 72.0%-84.0%; *p*-value <0.01) (Table2). The effectiveness of any (one or more) doses of Euvichol-plus vaccine was 74% (95% CI= 50.0-86.0%; p-value <0.01) (Table 3).

Table 3: Effectiveness of Euvichol vaccination

Number of doses	Cases (n=79)	Controls (n=316)	AOR* (95% CI)	VE	P-value
Any dose	51	268	0.26 (0.14-0.50	74.0% (50-86)	<0.01
2	10	189	0.19 (0.16-0.28)	81.0 (72-84.0)	<0.01

VE=vaccine effectiveness

*Adjusted for age, education, place of residence

For peer review only - http://bmjopen.bmj.com/site/about/guidelines.xhtml

DISCUSSION

The aim of this study was to determine the effectiveness of Euvichol-plus vaccine administered during a mass OCV campaign as part of control measures following a cholera outbreak in Lusaka. Our findings show that two doses of Euvichol-plus vaccine confer effective protection against cholera and can thus serve as an intervention in the control of cholera outbreaks.

These findings add to the existing evidence on the effectiveness of a two-dose regimen of OCV. They are also consistent with previous studies, for example, a randomised control trial conducted in the Philippines [16] comparing Euvichol and WC-rBS showed that the efficacy of two doses of Euvichol-plus vaccine was non-inferior to that of WC-rBS in adults (80% vs 74%) and children (91% vs 88%)[16]. Thus, our findings suggest that two doses of Euvichol-plus OCV administered during a mass vaccination campaign in response to a cholera outbreak confer effective protection. This finding is also in line with previous observational studies that showed that killed OCVs are protective in endemic and outbreak settings [19,20]. In an effectiveness study of mass OVC conducted in Beira, Mozambique, Lucas and colleagues [21] found that two doses of WC-rBS vaccine conferred 78% (95% CI: 29-92%) protection when administered during the outbreak. These findings are also consistent with those reported from Tanzania where the WC-rBS vaccine conferred a protection of 79% (95% CI: 47-92%) when administered in cholera endemic areas of Zanzibar [22]. Similarly, two doses of BivWC OCV was found to be effective when deployed in response to cholera outbreaks and areas of high endemicity in Haiti, India, and Guinea, where they conferred 63% (95% CI: 8%–85%), 69% (95% CI: 15-89%), and 87% (95% CI: 57-96%), respectively [23].

Our findings also show that administration of any dose (one or two) conferred significant protection against cholera. This finding is consistent with previous observational studies which reported the effectiveness of a single dose of WC-rBS vaccine [24,25]. In South Sudan, for example, a study showed that the short-term effectiveness of a single dose of WC-rBS vaccine was 87%, when administered during outbreaks. Similarly, a study conducted in Haiti reported a 79% effectiveness (95% CI: 43-93%) of one dose of OCV [26]

Despite these benefits, our study did not have enough power to determine the long-term effectiveness of the two doses, and thus, it is unclear how long these doses would confer protection against cholera. Nevertheless, available evidence shows that vaccine effectiveness wanes over time [26]. A systematic review on the protection against cholera from killed whole-cell OVCs conducted by Bi and colleagues and published in the Lancet found that, although the average effectiveness of two doses of OCV at one

For peer review only - http://bmjopen.bmj.com/site/about/guidelines.xhtml

year post vaccination was 83%, it decreased to 69% after 2 years. Another study comparing the long-term vaccine effectiveness of two doses against one dose of OCV conducted in Haiti reported that two doses have a significantly higher long-term effectiveness than one dose [25]. It further showed a 76% protection up to four years for two dose vaccine [26]. On the contrary, these authors found that the 79% effectiveness of one dose of OCV observed at 1 year was completely diminished after two years. Thus, these findings clearly show the benefits of administering two doses of OCV. However, further research employing longitudinal study design is required to determine the long-term effectiveness of two doses of OCV in the local context.

Potential limitations of this study should be noted. First, our study findings on the effectiveness of two doses are based on a small sample (10 cases and 189 controls) that received two doses of the Euvichol-plus vaccine. Thus, the study could have been underpowered. Second, our OCV effectiveness was based on an observational epidemiological study design following a reactive vaccination campaign mounted in response to a cholera outbreak; evidence from observational studies is considered relatively weak. Third, the vaccine exposure status was based on participant self-reports and cards for both cases and controls. This could have introduced information bias and misclassification. In addition, selection of cases from the line list of all suspected cholera patients treated in various CTCs between January and June 2018 might not have represented cholera cases that did not seek care at a health facility.

Despite these limitations, our findings are important as they add to the current evidence on the effectiveness of a two-dose regimen of OCV in the control of cholera outbreaks. In addition, our use of a matched case-control study design, collection of data shortly after the OCV campaign and conditional regression analysis all minimized confounding and increased validity of the study findings. Moreover, selection of cases from the line list with confirmed diagnosis reduced misclassification bias, making the study findings valid.

CONCLUSION

Our findings show that two doses of Euvichol-plus vaccine are effective in the control of the cholera outbreak in Lusaka, Zambia. The findings also show that two doses of the vaccine are more effective than a single dose. These results provide the evidence for and support the use of two doses of the vaccine as part of an integrated intervention to cholera control during outbreaks. Nevertheless, it is unclear how long these doses would confer protection against cholera and the long-term effectiveness

For peer review only - http://bmjopen.bmj.com/site/about/guidelines.xhtml

of the two doses is not well understood. Longitudinal studies are required to determine the long-term effectiveness of two doses of the OCV among the vaccinated populations in the local context. Further research is also required to determine the effectiveness and usefulness of Euvichol-plus vaccine in conferring herd immunity among non-vaccinated individuals during mass immunization and to determine the required minimum coverage. Finally, further research is needed to determine Euvichol-plus vaccine effectiveness among people living with HIV and its usefulness among these populations.

Figure Legend/Caption

Figure 1 : Participant recruitment algorithm

Contributorship

VM, CS, OC, BM, ANM, BG, WN, MK,LM, and MK contributed to the conception of the study, literature search, data collection as well as the drafting of the manuscript including data analysis, figures and results interpretation. KM, CM, NS, PB, KZ, LM and MM contributed to the coordination of the field study activities. VM, BM, NB, KM, CM contributed to the coordination of conception of the study and the literature search. CS, MK, BM, WN, MM, NB, contributed to the revision of the manuscript. All authors read and approved the manuscript.

Funding Statement

Ministry of Health and World Health Organization

Competing Interests

All authors declare that they have no conflicting interests in this work

Ethics approval

Ethical approval for the study was obtained from the Excellence in Research Ethics and Science (ERES, 2019-09-001) Converge Committee and authority to conduct the study was obtained from the National Health Research Authority. Written informed consent was obtained from all participants in the study.

Data Sharing

Data are available upon reasonable request from the corresponding author and with permission of the ERES ethics review board.

For peer review only

For peer review only - http://bmjopen.bmj.com/site/about/guidelines.xhtml

Costs and payments

There was no cash payment provided to participants for any portion of the study. Participants volunteered their time taken to participate in the study. As a small token of appreciation for their time and opportunity costs, participants received a bottle of water or soft drink, in line with the ERES guideline.

Acknowledgements

We thank Lusaka District Health Management Team who provided field research assistants and study participants. The GTFCC supported the country with OCV stocks and operations funds.

For peer review only

References

1. WHO, 2022. Cholera. Available at: https://www.who.int/news-room/fact-sheets/detail/cholera, accessed on 10th July, 2022
2. Ali M, Lopez AL, You YA, Kim YE, Sah B, Maskery B, Clemens J. The global burden of cholera. Bull World Health Organ. 2012;90(3):209-218A. doi: 10.2471/BLT.11.093427. Epub 2012 Jan 24. PMID: 22461716; PMCID: PMC3314202
3. Mwaba J, Debes AK, Shea P, Mukonka V, Chewe O, Simuyandi M et al. Identification of cholera hotspots in Zambia: A spatiotemporal analysis of cholera data from 2008 to 2017. PLoS Negl Trop Dis 14(4): e0008227. https://doi.org/10.1371/journal.pntd.0008227
4. Olu O, Babaniyi O, Songolo P, Matapo B, Chizema E, Kapin'a-Kanyanga M, Musenga E, Walker O. Cholera Epidemiology in Zambia from 2000 to 2010: Implications for improving Cholera prevention and control strategies in the country. East Afr Med J. 2013;90(10):324-31. Pmid: 26862642.
5. WHO, 2018. Zambia cholera outbreak situation reports available at: https://www.afro.who.int/countries/zambia/situation-reports/cholera, accessed on 10th July, 2022

For peer review only - http://bmjopen.bmj.com/site/about/guidelines.xhtml

6. N. Sinyange *et al.*, 'Cholera Epidemic - Lusaka, Zambia, October 2017-May 2018', *MMWR Morb. Mortal. Wkly. Rep.*, vol. 67, no. 19, pp. 556–559, May 2018

7. World Health Organization, 2018. Regional framework for the implementation of the global strategy for cholera prevention and control, 2018–2030: Report of the Secretariat. Regional Office for Africa. Regional Committee for Africa, 68. Available at: . https://apps.who.int/iris/handle/10665/275121, accessed on 10th July, 2022

8. WHO, 2017. Cholera vaccines: WHO position paper – August 201. Available at: https://www.who.int/publications/i/item/who-wer9234-477-500, accessed on 10th July, 2022

9. Bi Q, Ferreras E, Pezzoli L, Legros D, Ivers LC, Date K et al. Protection against cholera from killed whole-cell oral cholera vaccines: a systematic review and meta-analysis. Lancet Infect Dis. 2017;17(10):1080-1088. doi: 10.1016/S1473-3099(17)30359-6

10. Bhattacharya SK, Sur D, Ali M, Kanungo S, You YA, Manna B et al. 5 year efficacy of a bivalent killed whole-cell oral cholera vaccine in Kolkata, India: a cluster-randomised, double-blind, placebo-controlled trial. Lancet Infect Dis. 2013;13 (12):1050-6. doi: 10.1016/S1473-3099(13)70273-1.

11. Qadri F, Wierzba TF, Ali M, Chowdhury F, Khan AI, Saha A et al. Efficacy of a Single-Dose, Inactivated Oral Cholera Vaccine in Bangladesh', *N. Engl. J. Med.*, vol. 374, no. 18, pp. 1723–1732

12. Ferreras E, Chizema-Kawesha E, Blake A, Chewe O, Mwaba J, Zulu G et al. Single-Dose Cholera Vaccine in Response to an Outbreak in Zambia. N Engl J Med. 2018; 378(6):577-579. doi: 10.1056/NEJMc1711583. PMID: 29414267.

13. Luquero FJ, Grout L, Ciglenecki I, Sakoba K, Traore B, Heile M et al. Use of Vibrio cholerae vaccine in an outbreak in Guinea. N Engl J Med. 2014 May 29; 370 (22):2111-20. doi: 10.1056/NEJMoa1312680.

For peer review only - http://bmjopen.bmj.com/site/about/guidelines.xhtml

14. Ivers LC, Hilaire IJ, Teng JE, Almazor CP, Jerome JG, Ternier R et al. Effectiveness of reactive oral cholera vaccination in rural Haiti: a case-control study and bias-indicator analysis. The Lancet. Global health, 2015; 33: e162-8

15. Wierzba TF, Kar SK, Mogasale VV, Kerketta AS, You YA, Baral P et al. Effectiveness of an oral cholera vaccine campaign to prevent clinically-significant cholera in Odisha State, India. Vaccine, 2015; 15; 33 (21):2463-9. doi: 10.1016/j.vaccine.2015.03.073.

16. Verani JR, Baqui AH, Broome CV, Cherian T, Cohen C et al. Case-control vaccine effectiveness studies: Preparation, design, and enrollment of cases and controls. Vaccine. 2017 Jun 5;35(25):3295-3302. doi: 10.1016/j.vaccine.2017.04.037.

17. Central Statistical Office Zambia. 2010 census of population and housing. Available at: https://www.zamstats.gov.zm/phocadownload/2010_Census/2010%20Census%20of%20Population%20National%20Analytical%20Report.pdf, accessed on 1st July, 2022.

18. StataCorp (2015) Stata Statistical Software: Release 14. StataCorp LP, College Station, TX StataCorp LP. - References - Scientific Research Publishing. Available at: https://www.scirp.org/, accessed on 10th July, 2022

19. Azman AS, Parker LA, Rumunu J, Tadesse F, Grandesso F, Deng LL et al. Effectiveness of one dose of oral cholera vaccine in response to an outbreak: a case-cohort study. Lancet Glob Health, 2016; 4(11):e856-e863. doi: 10.1016/S2214-109X(16)30211-X. PMID: 27765293.

20. Baik YO, Choi SK, Olveda RM, Espos RA, Ligsay AD, Montellano MB et al. A randomized, non-inferiority trial comparing two bivalent killed, whole cell, oral cholera vaccines (Euvichol vs Shanchol) in the Philippines. Vaccine. 2015;33 (46):6360-5. doi: 10.1016/j.vaccine.2015.08.075.

For peer review only - http://bmjopen.bmj.com/site/about/guidelines.xhtml

21. Lucas ME, Deen JL, von Seidlein L, Wang XY, Ampuero J, Puri M et al. Effectiveness of mass oral cholera vaccination in Beira, Mozambique. N Engl J Med. 2005; 352(8):757-67. doi: 10.1056/NEJMoa043323.

22. Khatib AM, Ali M, von Seidlein L, Kim DR, Hashim R, Reyburn R et al. Effectiveness of an oral cholera vaccine in Zanzibar: findings from a mass vaccination campaign and observational cohort study. Lancet Infect Dis. 2012; 12(11):837-44. doi: 10.1016/S1473-3099(12)70196-2.

23. Song KR, Lim JK, Park SE, Saluja T, Cho SI, Wartel TA, Lynch J. Oral Cholera Vaccine Efficacy and Effectiveness. Vaccines (Basel). 2021;9(12):1482. doi: 10.3390/vaccines9121482.

24. Schwerdtle P, Onekon CK, Recoche K. A Quantitative Systematic Review and Meta-Analysis of the Effectiveness of Oral Cholera Vaccine as a Reactive Measure in Cholera Outbreaks. Prehosp Disaster Med. 2018 Feb;33(1):2-6. doi: 10.1017/S1049023X17007166.

25. Wierzba TF. Oral cholera vaccines and their impact on the global burden of disease, Human Vaccines & Immunotherapeutics, 2019; 15 (6): 1294-1301, DOI: 10.1080/21645515.2018.1504155

26. Franke MF, Ternier R, Jerome JG, Matias WR, Harris JB, Ivers LC. Long-term effectiveness of one and two doses of a killed, bivalent, whole-cell oral cholera vaccine in Haiti: an extended case-control study. Lancet Glob Health. 2018 Sep;6(9):e1028-e1035. doi: 10.1016/S2214-109X(18)30284-5.

For peer review only - http://bmjopen.bmj.com/site/about/guidelines.xhtml

Figure 1: Summary of participant selection process

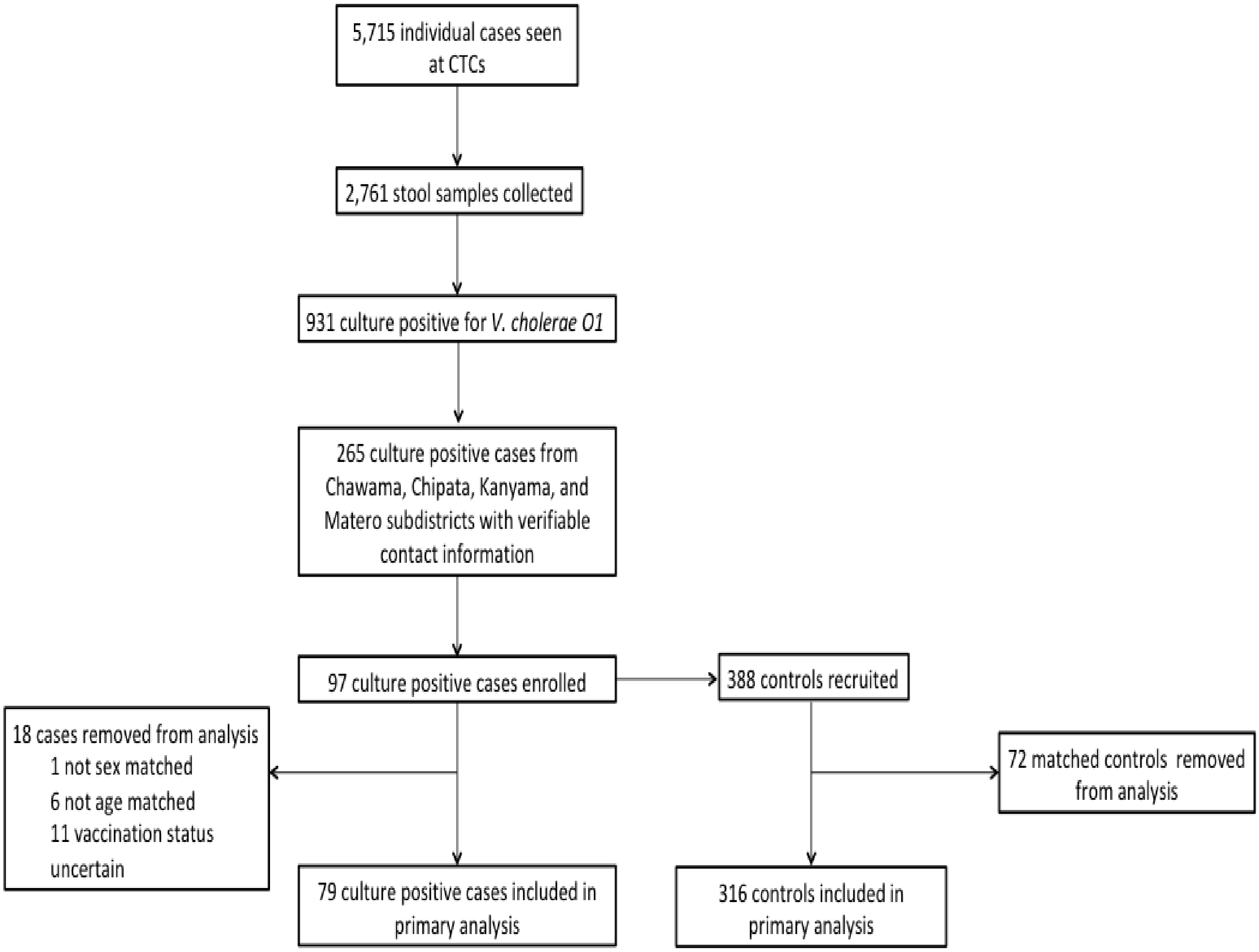

STROBE Statement—Checklist of items that should be included in reports of case-control Studies

**Item**	**Recommendation**	Page
Title and abstract	Indicate the study's design with a commonly used term in the title or the abstract	1
	Provide in the abstract an informative and balanced summary of what was done and what was found	2
Introduction		
Background/rationale	Explain the scientific background and rationale for the investigation being reported	4
Objectives	State specific objectives, including any pre-specified hypotheses	5
Methods		
Study design	Present key elements of study design early in the paper	5
Setting	Describe the setting, locations, and relevant dates, including periods of recruitment, exposure, follow-up, and data collection	5
Participants	Give the eligibility criteria, and the sources and methods of selection of participants. Describe methods of follow-up	5
Study size	Explain how the study size was arrived at	6
Variables	Clearly define all outcomes, exposures, predictors, potential confounders, and effect modifiers. Give diagnostic criteria, if applicable	7
Data sources/ measurement	For each variable of interest, give sources of data and details of methods of assessment (measurement). Describe comparability of assessment methods if there is more than one group	7
Bias	Describe any efforts to address potential sources of bias	8
Statistical methods	Describe all statistical methods, including those used to control for confounding	8
	If applicable, explain how loss to follow-up was addressed	
Patient and public involvement		8
Ethical considerations		9
Results		
Participants	Report numbers of individuals at each stage of study—eg numbers potentially eligible, examined for eligibility, confirmed eligible, included in the study, completing follow-up, and analysed (b) Give reasons for non-participation at each stage Participants (c) Consider use of a flow diagram	9
Descriptive statistics	(a) Give characteristics of study participants (eg demographic, clinical, social) and information on exposures and potential confounders<br>Descriptive data<br>(b) Indicate number of participants with missing data for each variable of interest	10

For peer review only

Outcome data	Report numbers in each exposure category, or summary measures of exposure	12
Main results		13
Discussion		13
Limitations		15
Conclusion		15
References		16

For peer review only
